# Should nurses be allowed to perform the pre-operative surgical site marking instead of surgeons? A prospective feasibility study at a Swiss primary care teaching hospital

**DOI:** 10.1186/s13037-017-0125-1

**Published:** 2017-04-04

**Authors:** Judit Schäfli-Thurnherr, Annette Biegger, Christopher Soll, Gian A. Melcher

**Affiliations:** 1 Department of Surgery, Hospital Uster, Uster, Switzerland; 2 Department of Visceral and Thoracic Surgery, Cantonal Hospit al Winterthur, Winterthur, Switzerland

**Keywords:** Surgery, Safety, Time-out, Implementation, Surgical site marking, Wrong-site surgery

## Abstract

**Background:**

Surgical site marking is one important cornerstone for the principles of safe surgery suggested by the WHO. Generally it is recommended that the attending surgeon performs the surgical site marking. Particularly in the case of same day surgery, this recommendation is almost not feasible. Therefore we systematically monitored, whether surgical site marking can be performed by trained nursing staff.

The aim of the study was to find out whether surgical site marking can be carried out reliably and correctly by nurses.

**Methods:**

The prospective non-controlled interventional study took place in a single primary care hospital of Uster in Switzerland. During a pilot phase of 3 months (starting October 2012) the nursing staff of a single ward was trained and applied the surgical site marking on behalf of the responsible surgeon. After this initial phase the new concept was introduced in the entire surgical department. 12 months after the introduction of the new concept an interim evaluation was performed asking whether the new process facilitates daily routine and surgical site marking was performed correctly. 22 months after the introduction a prospective data collection monitored for one month whether the nursing staff carried out surgical site marking independently and correctly. Data were collected by a patient-accompanying checklist that was completed by the nursing staff, the staff in the operating room and the responsible surgeons.

**Results:**

The stepwise implementation of the new concept of surgical site marking was well accepted by the entire staff. 150 patient-accompanying checklists were analyzed. 22 data sheets were excluded from the analysis. 90% (*n* = 115/128) of the surgical site markings were correctly performed. For the remaining 10% either a surgical site marking was not necessary or the nursing staff asked a surgeon to mark the correct surgical site. During the whole study time of almost 3 years, no wrong-site surgery occurred.

**Conclusion:**

Surgical site marking can be performed by trained nurses. However, the attending surgeon remains fully responsible of the correct operation on the correct patient.

## Background

Wrong-site surgery (WSS) is a complication with potentially devastating effects [1, 2]. The incidence is estimated at 1 in 30.000 to less than 1 in 100.000 surgeries [3, 4]. But the true incidence might be higher due to a reporting bias. In 2008 the WHO introduced the safe surgery checklist with the aim to reduce mistakes in patient care and adverse events by improving teamwork and communication [5, 6]. Many hospitals worldwide accepted the principles of safe surgery and introduced, inter alia, a team time-out before and after surgery. One important cornerstone for eradicating WSS is the surgical site marking before the intervention [7, 8]. The introduction of surgical safety checklists improved markedly the surgical outcome [9].

There is a large variance in the adherence to the principals of safe surgery and the use of checklists [8, 10, 11]. Generally, it is recommended that the attending surgeon himself marks the surgical site on the patient before surgery [6, 12–14]. In everyday practice with same day surgery this is often impossible due to a crowded schedule in the operating room. The time available between patient’s entry at the surgical ward, pre-medication and transfer to the operating room is often kept very short to avoid waiting time, especially in efficiently organized outpatient or same day surgery. Hence, it is often not possible that the attending surgeon or a resident marks the surgical site of the patient. It is also not reliable and responsible that the patient participates in the surgical site marking [15].

Therefore we asked whether surgical site marking could be assigned by trained nursing staff at the admission without affecting patient’s safety.

## Methods

**Fig. 1:**
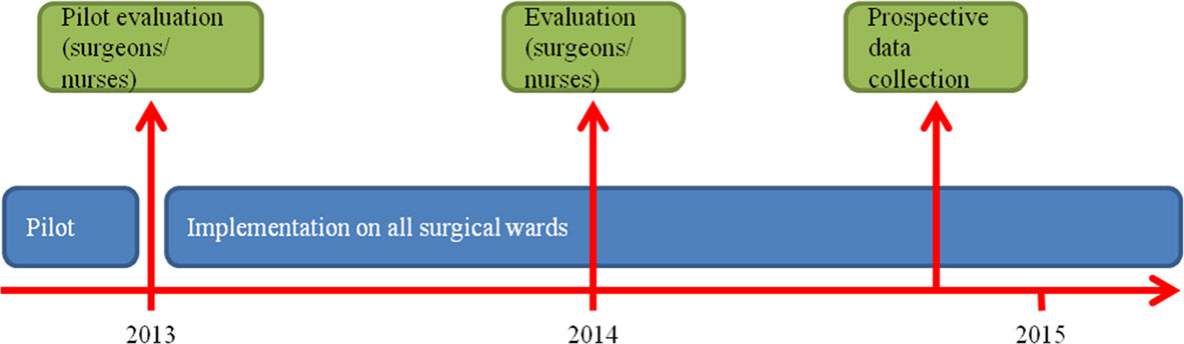
Timeline of the study with consecutive evaluations

The prospective non-controlled interventional study took place from 2012 until 2015 at the clinic for general and orthopedic surgery in the primary care hospital of Uster in Switzerland. The hospital of Uster is a public teaching hospital with a catchment area of app. 200.000 inhabitants with 5.000 in-patients and 19.000 out-patients per year in all surgical unities. The different surgical specialties include orthopaedic surgery, general and trauma surgery, abdominal surgery, hand surgery and urology. The hospital follows the guidelines of the WHO surgical safety checklist. Before 2012 the attending surgeon or the surgical resident assigned surgical site marking. A timeline of the study is given in Fig. 1.

### Phase 1: pilot

During 3 months (August 2012–October 2012) the nursing staff on a single surgical ward applied the pre-operative site marking and completed a patient-accompanying checklist regarding the upcoming intervention. All participating nurses were trained regarding the correct surgical site marking.

### Phase 2: implementation

After the pilot phase, the process was established on all surgical wards of the clinic in January 2013. Twelve months after implementation a questionnaire-based first evaluation of the new process was done asking nursing staff and physicians about feasibility and satisfaction about the new process. **Quality control:** 22 months after implementation an additional questionnaire determined whether the surgical site marking was attached independently, whether uncertainties existed regarding the marking, or whether a surgeon was called to label the surgical site correctly. This questionnaire was filled in simultaneously by the surgeon in charge, the nurses applying the surgical site mark, and the staff of the OR. Data were collected from October 15^th^ until November 15^th^ 2014. During the team time-out shortly before surgery, the attending surgeon recorded on the checklist whether the site marking was done properly. All surgical site markings were recorded prospectively with a patient-accompanying checklist and questionnaire. Emergencies were excluded from data collection.

## Results

**Fig. 2:**
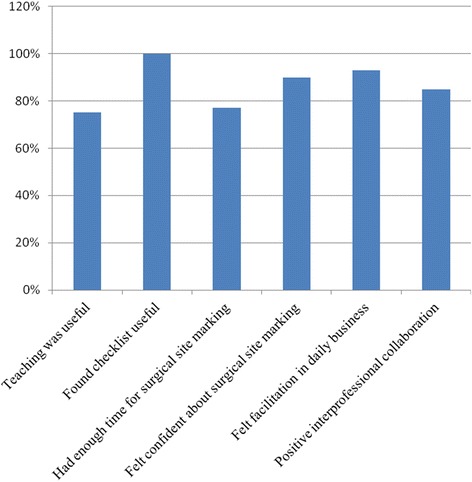
Evaluation of the nursing staff after pilot phase showing acceptance of the new process

After closing the pilot phase, a written evaluation filled in by 13 participating nurses showed a positive attitude towards the new process and a significant benefit in the daily business (Fig. 2). Thereafter, the new process was implemented to the entire surgical department.

**Fig. 3:**
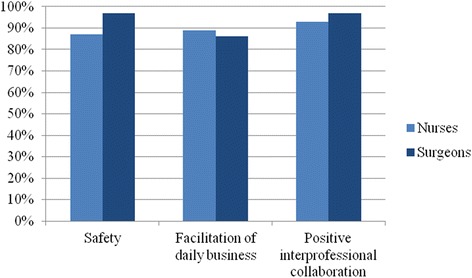
Evaluation 12 months after implementation showing a corresponding perception of the new process

Twelve months after implementation 50 members of the nursing staff were asked whether they felt confident to place the surgical site marking and whether the process facilitated daily routine. 40 surgeons were asked whether surgical site marking was performed correctly and reliably by the nursing staff (Fig. 3).

Eighty-seven percent of the nursing staff felt confident to place the surgical site marking and 89% mentioned that the process facilitated daily routine. Almost all surgeons (97%) trusted the surgical site marking done by the nursing staff and 86% of the surgeons noticed facilitation. Both surgeons and nursing staff felt a positive side effect in inter-professional collaboration (Fig. 3).

**Fig. 4:**
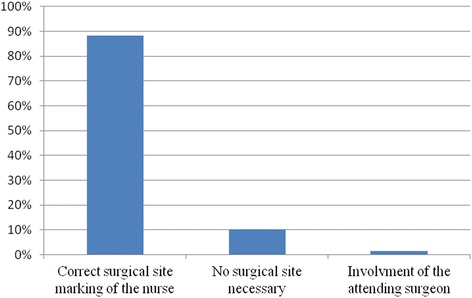
Prospective data collection 22 months after implementation

Twenty two months after implementation a prospective data collection took place. During one month 353 patients for elective surgery were recorded and surgical site marking was performed by the nursing staff. The return rate of the patient-accompanying checklist and questionnaires was 42% (*n* = 150/353). Twenty two data sheets were incomplete and therefore were excluded from the analysis. One hundred twenty-eight data sheets were available for analysis (Fig. 4). In 90% (*n* = 115/128) surgical site markings were correctly performed. In only two cases (1.7%; *n* = 2/115) the nursing staff were not able to perform the site marking and asked a surgeon to mark the patient. In 10% (*n* = 13/128) surgical site marking was performed although it was not necessary (e.g., laparoscopic cholecystectomy or rectosigmoid resection). Analysis of the team-time out before surgery indicated that surgical site marking was done correctly in all of the 353 patients and no WSS occurred during the study period.

## Discussion

The study shows that surgical site marking can be assigned safely by the nursing staff at the patient’s admission. Due to a stepwise implementation the new process was introduced without difficulties. All surgical site markings were performed properly and on the correct side. The evaluation of the modified process of surgical site marking was done by the nursing staff and surgeons. Both groups noticed a facilitation of daily routine work.

Although the return rate of the data sheets was only 42%, no wrong site surgical site marking occurred during the time of the study. Apparently, there was a non-compliance regarding the completion of the questionnaires for the study but not of the surgical site marking. The reason may have been lack of time during the workday or a general lack of interest in surveys. We did not insist on full completion of the questionnaires to the staff to avoid a response bias. However, the questionnaires indicate the daily workload was reduced for surgeons and nursing staff. On the one hand the nursing staff does not have to organize or wait for a surgeon during the admission process, especially in the case of same day surgery. On the other hand the surgeon does not have to interrupt daily routine by e.g., changing clothes from the operation room and walk to the admission ward.

A central criticism of the concept might be that the surgeon distances himself from the patient. Without signing one’s site before surgery the opportunity is lost to engage the patient and to ease anxiety regarding the upcoming surgery [16, 17]. But in the setting of same day surgery the surgeon meets the patient in advance in the outpatient clinic to discuss the risks and benefits of the intervention. The outpatient clinic provides an undisturbed and quiet environment to discuss all aspects within an adequate time frame.

We also asked lawyers from three different institutions, whether tasks like surgical site marking could be performed on a legal basis in Switzerland by the nursing staff. The involved institutions were a) Swiss Foundation for Patient Safety b) Swiss Professional Association of Care and c) Cantonal Medical Service of Zurich. All three lawyers agreed that surgical site marking can be delegated to the nursing staff. However, the surgeon remains fully responsible for the correct intervention.

The results of the study also suggest that the implementation of the principles of safe surgery including safety checklists and surgical site marking are more important than the question of who should perform surgical site marking. Although the surgeon is responsible for his intervention, all staff has to carry out the principles of safe surgery to avoid mistakes [18, 19].

## Conclusion

Based on this study we have introduced surgical site marking during the admission process as a task of the nursing staff and hereby facilitated the daily routine of surgeons and nurses, especially in the setting of same day surgery. However, as wrong site surgery is a very rare surgical event, future large-scale studies are necessary to strengthen the results of our study. It should be emphasized that every practising surgeon remains fully responsible of performing the correct operation on the correct patient.

## Abbreviations

OR: Operating room
WHO: World health organization
WSS: Wrong site surgery
